# Mouse genetic approaches applied to the normal tissue radiation response

**DOI:** 10.3389/fonc.2012.00094

**Published:** 2012-08-07

**Authors:** Christina K. Haston

**Affiliations:** Meakins-Christie Laboratories and the Department of Medicine, McGill UniversityMontreal, QC, Canada

**Keywords:** radiotherapy, radiation toxicity, murine strain differences, genetic variation, lung, skin, intestine

## Abstract

The varying responses of inbred mouse models to radiation exposure present a unique opportunity to dissect the genetic basis of radiation sensitivity and tissue injury. Such studies are complementary to human association studies as they permit both the analysis of clinical features of disease, and of specific variants associated with its presentation, in a controlled environment. Herein I review how animal models are studied to identify specific genetic variants influencing predisposition to radiation-induced traits. Among these radiation-induced responses are documented strain differences in repair of DNA damage and in extent of tissue injury (in the lung, skin, and intestine) which form the base for genetic investigations. For example, radiation-induced DNA damage is consistently greater in tissues from BALB/cJ mice, than the levels in C57BL/6J mice, suggesting there may be an inherent DNA damage level per strain. Regarding tissue injury, strain specific inflammatory and fibrotic phenotypes have been documented for principally, C57BL/6 C3H and A/J mice but a correlation among responses such that knowledge of the radiation injury in one tissue informs of the response in another is not evident. Strategies to identify genetic differences contributing to a trait based on inbred strain differences, which include linkage analysis and the evaluation of recombinant congenic (RC) strains, are presented, with a focus on the lung response to irradiation which is the only radiation-induced tissue injury mapped to date. Such approaches are needed to reveal genetic differences in susceptibility to radiation injury, and also to provide a context for the effects of specific genetic variation uncovered in anticipated clinical association studies. In summary, mouse models can be studied to uncover heritable variation predisposing to specific radiation responses, and such variations may point to pathways of importance to phenotype development in the clinic.

## Introduction

Radiation therapy, which is frequently used in the treatment of cancer (Barnett et al., 2009), produces pathologies of excessive inflammation or deposition of extracellular matrix in the normal tissue interstitium (fibrosis) which is necessarily also exposed. If excessive, this damage can result in impaired tissue function (Carver et al., 2007). Clinically, pretreatment knowledge of patients likely to develop these side effects and those who are not would be useful in prescribing this modality. Additionally, knowledge of genetic variation associated with the likelihood of developing severe tissue responses following unplanned radiation exposures may influence the assignment of medical intervention to a population.

A person's response to radiation is dictated by dose, tissue volume treated, and by yet unknown inherent factors or genetic predisposition to adverse effects. The hypothesis that there is a genetic basis for susceptibility to radiotherapy associated normal tissue response is being investigated, clinically, by international consortia called Genepi and RAPPER (Barnett et al., 2009). These groups are analyzing single nucleotide polymorphism (SNP) variation in collated populations of ~3000 patients for whom radiation adverse responses have been recorded in various tissues including breast, lung, and the gastrointestinal tract. This work is likely to reveal associations of particular genes with radiation-induced side effects, but may not inform of mechanism as, for most of genome, gene function is still to be determined. Further, the identification of causal genetic variations influencing normal tissue response using clinical data can be confounded by the effects of multiple genes and their interactions on the phenotype, and by the cancer and other therapies such that complementary approaches in controlled model systems are warranted. Studies in mouse models can circumvent the difficulties presented by clinical association studies as sufficient numbers of genetically defined offspring, in a controlled environment, can be produced (Hunter, 2012).

Herein I present how mouse inbred strain data can be used in the investigation of genetic basis of a trait; focusing on radiation-induced tissue injuries in the lung, skin, and intestine. Many important radiation-induced clinical responses differ in presentation among inbred strains, including variation in the primary injury (DNA damage, repair, cell death) and in the tissue response to this primary injury (inflammation and fibrosis). These differences permit the identification of potentially novel genes, which harbor variants contributing to the trait, to be achieved through mapping or association studies. Among the strengths of mapping studies is that these are completed without assumptions regarding which genes are involved in the phenotype. It is anticipated that the mouse studies will provide evidence for variation within specific genes which predisposes to specific normal tissue responses, and that these genes could then be investigated as contributing to the same responses clinically. The genetic evaluation of inbred strain differences also enables the assessment of the effect of clinically identified risk alleles in the context of known, both genetically and by phenotype, backgrounds (Andreassen, 2010). In this regard studies in mouse models are anticipated to make an important contribution as genetics advances from variant identification to variant interpretation (Barnett et al., 2009; Cooper and Shendure, 2011; Hunter, 2012).

## Variation in primary response to radiation

Interindividual variation in primary radiation responses has been documented in both human and mouse populations. Using immortalized B cells derived from members of Centre d'Étude du Polymorphisme Humain Utah pedigrees, Smirnov et al. (2009) established individuals to differ in radiation-induced changes in gene expression; and identified regions of the genome to be linked to these changes. Secondly, by measuring the survival post irradiation of human-derived lymphoblastoid cell lines Niu et al. (2010) identified there to be inherent variation in this trait and also identified genetic variation associated with the phenotype. Thus there is clear evidence that the responses of cells to irradiation, assessed by gene expression and survival, vary from person to person.

In mice, the primary radiation response of DNA damage, indicated by gamma H2AX foci, has also been shown to be strain dependent. For example, Bhogal et al. (2010) reported the numbers of foci in skin at 7 days following total body irradiation exposure to be highest in BALB/cJ mice, followed by levels in the C3H/HeJ and C57BL/6J strains in this order. Rübe et al. (2008) also determined the numbers of radiation-induced gamma H2AX foci to depend on strain as levels in blood lymphocytes of BALB and C57BL/6J mice differed, again with greater numbers in BALB/cJ than C57BL/6J. Interestingly, this strain difference in DNA damage was also evident in different tissues including the intestine and lung (Rübe et al., 2008) suggesting there may be an inherent DNA damage level per strain. One question not yet addressed in such investigations is whether interindividual differences in the primary radiation responses of DNA damage, gene expression or cell survival dictate the tissue response to injury, but the integration of early and late assays of response in the same mouse models could determine whether such a relationship exists.

## Post-irradiation tissue repair

### Strain dependent radiation induced lung disease: murine phenotype mimics clinical

The radiotherapy associated lung injury is thought to occur through the ionizing radiation producing reactive oxygen species which induce lesions in DNA leading to damage of the alveolar epithelium (Osterreicher et al., 2004) and capillary endothelium (Van der Meeren et al., 2003). The inflammatory reaction to cell damage, including lymphocyte (Roberts et al., 1993) and neutrophil (Takigawa et al., 2000) recruitment, if excessive or sustained, likely through epithelial (Rube et al., 2005) and inflammatory cell (Roberts et al., 1993) derived cytokine signaling, can lead to the overwhelming inflammatory response of alveolitis/pneumonitis. The tissue response to injury can also be resolved as fibrosis, which is characterized by the progressive accumulation of extracellular matrix constituents replacing normal functional parenchyma (Kuwano et al., 2004) and may feature bone marrow cell recruitment (Epperly et al., 2003; Abe et al., 2004). Finally, the initial radiation insult could be insufficient to generate the cascade of response, or may be resolved without fibrotic scarring.

The three clinical outcomes of thoracic cavity radiotherapy (with respect to adverse effects) are fibrosis, alveolitis, or a subclinical response, and the times at which they occur (generally 3 months post therapy for alveolitis, 6 months for fibrosis; Carver et al., 2007) are each represented in the radiation response of an inbred mouse strain. We (O'Brien et al., 2005; Haston et al., 2007) and others (Sharplin and Franko, 1989a,b) have reported murine strain differences in response to radiation wherein following high dose whole thorax irradiation, C3H/HeJ mice develop a diffuse lethal alveolitis, C57BL/6J mice respond to lung irradiation with fibrosis and mice of certain recombinant congenic (RC) strains present a minimal lung response at 6 months post treatment (Lemay and Haston, 2008). The inflammatory responses of inbred mice also show similarities to that which has been reported for clinically. In particular, the alveolitis phenotype of increased mast cells has been identified to be a feature of clinical radiation-induced lung disease (Majori et al., 2000).

Genetically modified animals have been studied to investigate how particular genetic variations affect the radiation response of the lung. The principal phenotypes evaluated have been time to onset of distress, and the histological indication of alveolitis or fibrosis which contributed to the distress. In one such study, Travis et al. (2011) demonstrated that nuclear factor, erythroid derived 2, like 2 (*Nfe2l2*) deficient mice die earlier than wildtype mice, following whole thorax irradiation but that the genetic deficiency did not alter the amount of pulmonary fibrosis in distressed mice. With a similar experimental approach, integrin αvβ6−/− mice were shown to be completely protected from fibrosis, following radiation to the lung, but not from late radiation-induced mortality (Puthawala et al., 2008). Further, Yang et al. (2011) showed post thoracic irradiation survival to be enhanced in cc chemokine ligand 3 deficient (*Ccl3*−/−) mice or in mice deficient for receptor *Ccr1*−/−, relative to wild type, and for this response to be coincident with fewer lymphocytes and macrophages in lung tissue from the knockout mice. Finally, we reported the lung phenotype of toll-like receptor-4 (*Tlr4*)-deficient and *Tlr2*-deficient mice to not differ from that of wild type mice in terms of survival time post-irradiation, or by histological evidence of alveolitis or fibrosis. In contrast, mice deficient in both receptors developed respiratory distress at an earlier time than did wild type mice and presented an enhanced fibrotic response (Paun et al., 2010a). Given the established functions of *Tlr2* and *Tlr4* in innate immunity (Lee et al., 2012), *Ccl3* and *Ccr1* in lymphocyte recruitment (Tregoning et al., 2010), integrins in cell signaling (Campbell and Humphries, 2011) and Nfe2l2 in oxidative stress (Boutten et al., 2010), these data implicate components of these pathways in the development of radiation-induced lung injury.

Studies of genetically modified mice have revealed that the clinically important consequences of radiation-induced alveolitis and pulmonary fibrosis can be dissected in this animal model, but that many genes, and potentially distinct pathways, influence the development of these traits. Whether there are naturally occurring variations, of prevalence in the patient population, such that these genes (*Ccr1* or *Tlr2* and *4* for example) may influence the clinical response remains to be determined.

### Strain-dependent contribution to radiation-induced skin disease

With similarities to the two phase radiation response of the lung (Zhao and Robbins, 2009; Williams et al., 2010), the irradiated skin injury includes an inflammatory and fibrotic response depending significantly on radiation dose and area of skin involved (Guipaud et al., 2007; Kiang et al., 2010; Thanik et al., 2011). The phenotypes developing in radiation-induced skin injury consist of erythema, dry and moist desquamation, necrosis and fibrosis in both mice (Guipaud et al., 2007; Holler et al., 2009), and in clinic (Yoshida et al., 2010).

As with lung disease, the same radiotherapy regimen is known to produce individual variation in the skin telangiectasia response (Turesson, 1990), and this variability also occurs among inbred mouse strains (Iwakawa et al., 2003; Noda et al., 2005). Indeed, Iwakawa et al. (2003) showed 50 Gy to the leg produced the same peak skin reaction at 20 days post treatment in A/J, C57BL/6J and C3H/HeJ mice, but, interestingly, that the mouse strains differed in rate of repair of this injury as at day 70 the skin injury score of A/J mice was still high, C57BL/6J mice had a mid-range score while the skin of C3H/HeJ mice was normal by this time. In this model, therefore, the strain difference is in the tissue response to injury.

### Strain-dependent contribution to radiation-induced intestinal disease

Radiation-induced intestinal disease has also been successfully modeled in mice (Followill et al., 1993; Okoshi et al., 2008) permitting informative strain variation investigations and the evaluation of genetic modifications on trait development. Firstly, important investigations in a mouse model have revealed dose dependent responses to develop in the intestines of C3Hf/Kam mice wherein the extent of early crypt depletion dictated the risk of obstruction (Followill et al., 1993). The similarities of the mouse model to the clinical condition include the presence of inflammation and fibrosis, and the incidence of obstructions (Kountouras and Zavos, 2008). As with other tissues there is an inbred strain dependence in radiation response. For the intestine, strains differ in incidence of obstructions after a particular dose, which is greater in C57BL/6J, than C3H, mice (Skwarchuk and Travis, 1998a), and in the histological presentation of the injury, where C57BL/6J mice have more fibrosis in the lamina propria (Skwarchuk and Travis, 1998b). More recent studies with genetically altered mice have confirmed the radiation-induced intestinal disease manifestation to be due to the death of epithelial cells and have further shown these cells to die by a mechanism that is regulated by p53 but which is independent of apoptosis (Kirsch et al., 2010) and that a specific crypt intestinal stem cell population is responsible for crypt and villi regeneration following radiation injury (Yan et al., 2012).

Mouse models of primary response and tissue injury reflect that measured in the clinic both histologically and with variability among individual persons and individual strains. The injuries of inflammation/fibrosis may indicate a common tissue repair response. Despite the documentation of strain specific radiation-response phenotypes, and mechanistic similarities in their development, a correlation among phenotypic responses such that knowledge of the radiation injury in one tissue informs of the response in another is not evident. Investigations in larger numbers of inbred strains will be needed to definitively address this possibility.

## Genetic mapping in mice

The strain-dependent phenotypes measured in mice can serve as the base of genetic studies which aim to reveal the set of (strain-dependent) genetic variation which influences the phenotype development. A set of genes is indicated as radiation-induced injuries are genetically complex traits, wherein variation within more than one gene affects the phenotype, and are thus controlled by the cumulative actions of alleles at multiple loci. Toward identifying these genes, investigators first define the region of the genome linked to the phenotype, in a study called a quantitative trait locus (QTL) mapping study. In such studies progeny derived from the inbred strains which differ in the trait of interest are generated, phenotyped and genotyped. Statistical analyses reveal the region(s) of the genome linked to the trait (Peters et al., 2007; Arends et al., 2010). A QTL is a region of the genome linked with a measurable phenotype (Peters et al., 2007), such as the percent of the lung with fibrosis, or the time to repair of a radiation-induced skin injury. Based on mapping studies in 150–200 progeny, and the size of the mouse genome, each QTL usually encompasses a genomic region of approximately 200 genes.

Three QTL mapping studies have been completed which reveal regions of the genome linked to the radiation-induced lung responses of pneumonitis/alveolitis and fibrosis (listed in Table 1). Additional radiation-induced tissue responses have not been mapped, but as indicated earlier genetic loci of radiation-induced changes in gene expression (Smirnov et al., 2009) and cell survival (Niu et al., 2010) have been reported. Regarding lung disease, in one study, the phenotypic difference in lung response between C57BL/6 (fibrosis) and C3H (alveolitis) responding strains was used to map loci of radiation-induced pulmonary fibrosis, named *Radpf1* on chromosome 17 and *Radpf2* on chromosome 1 (Haston et al., 2002). Secondly, we identified the genomic location of variants which influence susceptibility to radiation-induced alveolitis, and determined the relationship of alveolitis to the development of fibrosis, by completing genome wide genotyping, and survival analysis and histological phenotyping, of a cohort of mice also derived from C57BL/6 and C3H strains (Haston et al., 2007). In this second QTL mapping study linkage to *Radpf1* and *Radpf2* was replicated, supporting the concept that genetic variation in these regions affects the trait of radiation-induced pulmonary fibrosis. Loci of alveolitis were also mapped by exploiting the strain difference in onset of this trait, of approximately 12 weeks post irradiation in C3H mice and 22–24 weeks post irradiation in the C57BL/6 strain. These loci were distinct from the map locations of the linkage intervals for fibrosis, indicating separate genes to be involved in these traits. Given the separation in loci, the mice inheriting promoting alleles at these loci developed fibrosing alveolitis (i.e., both alveolitis and fibrosis) and the mice inheriting the protective alleles at the alveolitis regions were spared lung disease and lived to the end of the experiment. Further, the fibrosis response was only evident in mice which had also developed alveolitis, and inherited fibrosis-promoting B6 alleles at one or more of the *Radpf* loci, while mice inheriting pre-disposing fibrosis alleles did not develop fibrosis in the absence of alveolitis. As the combination of sufficient alveolitis development and inheritance of fibrosis promoting alleles was necessary for the radiation-induced response of fibrosis, these findings link an inflammatory response to the development of fibrosis. Whether alveolitis contributes to fibrosis development clinically is not known but Giotopoulos et al. (2007) have shown the risk of lung fibrosis to be increased in breast cancer patients who develop an early acute reaction (skin inflammation) to radiation, thus an interaction between the development of these responses is plausible.

**Table 1:** **Genetic modifiers of radiation-induced lung disease**.

	**Effect**	**Reference**
**KNOCKOUT STUDY**		
*Nfe2l2*	Knockout mouse presents earlier onset distress	Travis et al., 2011
α*v*β*6*	Knockout mouse presents reduced radiation-induced pulmonary fibrosis	Puthawala et al., 2008
*CCl3, Ccr1*	Knockout mice present delayed onset distress; reduced inflammatory cell count	Yang et al., 2011
*Tlr2,4*	Combined knockout mouse presents earlier onset distress; enhanced radiation-induced pulmonary fibrosis	Paun et al., 2010a
**LINKAGE STUDY**		
B6 × C3H F2: *Radpf1-3*	Loci wherein specific B6 or C3H alleles increase radiation-induced pulmonary fibrosis mapped	Haston et al., 2002
B6 × C3H backcross mice	Loci wherein specific B6 alleles increase radiation-induced pulmonary fibrosis; C3H alleles promote onset of respiratory distress mapped	Haston et al., 2007
B6 × AJ recombinant congenic strains	Loci wherein specific B6 alleles increase radiation-induced pulmonary fibrosis; A/J alleles promote alveolitis mapped	Lemay and Haston, 2008

The final quantitative trait loci of radiation-induced lung response were mapped using RC mice (Lemay and Haston, 2008). RC strains are lines of mice which have been derived from two inbred progenitors, such that each RC strain has a known, but unique, 12.5% donor genome in the recipient strain background. We have mapped loci of radiation-induced alveolitis and fibrosis susceptibility in a panel of RC strains bred to have either 12.5% C57BL/6J genes in the A/J strain background, or the reverse, 12.5% A/J genes in the C57BL/6J strain background. With this resource the C57BL/6J alleles involved in the susceptibility to pulmonary fibrosis were divided among strains of mice, which permitted the assessment of the effect of discrete C57BL/6J genomic regions on this phenotype. Enabling this study was the observation that A/J mice succumb to a mast cell rich alveolitis post thoracic irradiation in contrast to the fibrosis response of C57BL/6J mice, and thus loci of the increased mast cell phenotype and alveolitis were mapped in these mice in addition to fibrosis. Finally, by evaluating the RC mice we were able to identify strains that survive high dose thoracic radiotherapy, a trait since it is not presented by inbred strains evaluated to date makes these established RC lines an important resource for genetic studies.

Investigations of genetically mixed mice, such as the progeny derived for a mapping study or RC strains are also useful for revealing correlations among phenotypes which may provide mechanistic insight into the development of the trait. For example, by inflammatory cell type phenotyping we found the mice succumbing to respiratory distress (from alveolitis), in both of the mapping studies (Haston et al., 2007; Lemay and Haston, 2008), had high levels of mast cells in their lung tissue. Given that mast cell proteases 1, 2, 4 and 8 and mast cell chymase 1 are among the genes mapped to the putative alveolitis regions, it is possible that the genetic variant underlying alveolitis susceptibility at these loci is related to mast cell recruitment or activity. To investigate the correlation of mast cell numbers to alveolitis development we assessed the effect of an inhibitor of this cell type on radiation-induced lung disease. Imatinib (Gleevec) treatment was shown to significantly reduce the number of mast cells in the lungs of irradiated mice and to increase post irradiation survival time (to delay the onset of alveolitis) by an average of 23% (Thomas et al., 2010). The delay in onset of respiratory distress brought about by the mast cell inhibitor is similar to that of mice inheriting the protective (C57BL/6J) allele for this trait which supports variation in mast cell genes as contributing to radiation-induced alveolitis in mice.

A second approach to revealing informative subphenotypes is to measure the molecular phenotype through gene expression analysis. For example, to define the response to 18 Gy pulmonary irradiation in mice, at the expression level, and to identify pathways which may influence the alveolitis and fibrosis phenotypes expression profiling has been completed. In this work, pathway analysis revealed the expression of complement and of B cell proliferation and activation genes to distinguish fibrosis from alveolitis (Paun et al., 2010b) thus variants in genes of these pathways are viable candidates for the fibrosis trait. With similar approaches, gene expression profiling has also been used to reveal repression of cell adhesion to be part of the recovery process following bone marrow irradiation in mice (Zhang et al., 2011) and reduced oxidative metabolism to be a component of the proliferative response in intestine following radiation injury (Van Landeghem et al., 2012).

## QTL to the gene

Mapping of the loci influencing radiation response is the first step towards identifying the genetic variation which contributes to the trait. As each may contain on the order of 200 genes, sets of experiments are used to isolate the causal variation contributing to the trait. To build on linkage studies investigations in congenic mice and gene finding approaches such as single nucleotide and haplotype reviews, expression experiments and knockout or pathway inhibitor studies may follow.

In detail, to avoid extensive investigation of false positive loci, studies of congenic mice, which differ from an inbred strain at a particular defined locus, can be used to confirm or refute the influence of each QTL on a trait. To further investigate a QTL, a congenic strain, in which the QTL alleles from the “low” strain are bred into the “high” strain, or the reverse, can be created and phenotyped. Evidence for the influence of the QTL on the phenotype is provided if the phenotype of the congenic strain differs from that of the “high” strain. From these congenic mice, subcongenic mice, or lines with a defined reduced portion of the QTL, can be bred and phenotyped to reduce the size of a linkage interval, and thus the number of genes contained which are positional candidates for the trait.

Recent studies to identify quantitative trait genes, or the specific genetic variation underlying a QTL, have used composites of experiments in congenic and subcongenic mice, with candidate gene sequence variation and expression analyses, to support their identification. For example Tomida et al. (2009) reported ubiquitin-specific peptidase *Usp46* to be the quantitative trait gene for mouse immobile behavior (related to depression like behavior) in a specific tail suspension and forced swimming test. In this work the authors phenotyped several congenic and subcongenic mouse strains to narrow a QTL to 0.5 Mb. The four genes in this reduced region were evaluated for expression in the target tissue (brain) by *in situ* hybridization, and for variation in coding sequence by sequencing of DNA from the inbred and congenic lines. These analyses revealed one of the genes to contain a codon deletion and transgenic mice generated with the allele lacking this deletion were rescued from the phenotype. Similarly, Scherneck et al. (2009), in an investigation of obesity associated diabetes, used congenic mice to narrow a linkage interval to 10 genes and assessed each of these for tissue appropriate expression and parental strain DNA sequence variation. One gene for which an allele specific truncated mRNA was produced was identified and obese mice with this mutation crossed into their genomes presented an altered phenotype. Finally, Tan et al. (2010) showed genetic variation to contribute to Type 1 diabetes susceptibility through an evaluation of congenic mice combined with both sequence analysis of the genes mapping to the reduced linkage interval in parental and congenic mice and tissue appropriate expression of the candidate gene.

Studies to pinpoint the genetic variants which underlie loci of radiation-induced tissue injuries in the mouse, such as *Radpf1*, have not yet been completed, and remain a challenge for the field. Thus, at present, there is no naturally occurring inbred strain DNA polymorphism or variant which is known to alter susceptibility to radiation-induced tissue injury.

## Conclusions

The genetic differences among inbred strains of mice lead to their variable susceptibility to clinically important radiation-induced traits including DNA damage and levels of tissue injury. Such strains are thus a resource for determining whether molecular events including changes in gene expression or level of DNA damage and repair influence extent of tissue injury; or for the investigation of other pre-injury phenotypes, as called for by Travis (2007). One goal in normal tissue research is to employ the well defined mouse models in investigations to dissect the specific variation which regulates individual differences in radiation-induced responses, with the ultimate aim of enhancing the use of clinical radiotherapy. The identification, in mice, of potential genetic variants influencing predisposition to radiotherapy associated side effects would be an important base for therapeutic development and for subsequent clinical investigation. Such insight is needed, as the candidate gene approach to identifying risk of tissue adverse effects in clinical data has not yet revealed variation which produces a clinically relevant effect (Barnett et al., 2012). Inversely, mouse models provide the controlled background in which to evaluate the effects of clinically identified risk alleles on the radiation-induced trait. Although such genes and variants are not yet definitively described, the existence of characterized inbred strain models coupled with advances in methods for clinical association studies (both in sequencing technology and analysis) produces promising opportunities for advances in this area of personalized medicine.

### Conflict of interest statement

The author declares that the research was conducted in the absence of any commercial or financial relationships that could be construed as a potential conflict of interest.
